# Design and Vision-Based Calibration of a Five-Axis Precision Dispensing Machine

**DOI:** 10.3390/mi17010053

**Published:** 2025-12-30

**Authors:** Ruizhou Wang, Jinyu Liao, Binghao Wang, Qifeng Zhong, Yongchao Dong, Han Wang

**Affiliations:** State Key Laboratory of Precision Electronic Manufacturing Technology and Equipment, Guangdong University of Technology, Guangzhou 510006, China; 13423328413@163.com (J.L.); 13922961022@163.com (B.W.); qifeng6018@163.com (Q.Z.); dongych@gdut.edu.cn (Y.D.); wanghangood@gdut.edu.cn (H.W.)

**Keywords:** geometric error identification, kinematics calibration, vision-based measurement (VBM), five-axis dispensing machine

## Abstract

Five-axis precision dispensing machines are employed for semiconductor packaging. The dispensing accuracy is significantly affected by multiple geometric errors among the five axes. This paper proposes a vision-based measurement (VBM) system for identifying geometric errors and calibrating kinematics. The VBM system is also employed to complete the detection of the workpiece. A kinematic model of the machine was established using a local product-of-exponential formulation of screw theory. A geometric error identification algorithm was designed. Eight position-independent geometric errors (PIGEs) and position-dependent geometric errors (PDGEs) were involved. The system of overdetermined equations was solved. Combining the singular value decomposition and regularization, eight PIGEs in the A and C axes were identified. Comprehensive error measurement results verified the proposed approach. The VBM system measured a mean spatial position error of approximately 59.9 μm and a mean orientation error of about 160 arcsec for the end-effector, reflecting the geometric error level of the prototype machine. The proposed approach provides a feasible and automated calibration solution for five-axis precision dispensing machines.

## 1. Introduction

Precision dispensing is a critical step in semiconductor packaging processes. The task imposes increasingly stringent requirements on dispensing position accuracy and motion trajectory stability amid the rapid evolution of display technologies [1]. Five-axis dispensing machines leverage multi-axis coordinated spatial pose control capabilities [2]. The machine has been adopted in precision scenarios that require precise control over adhesive morphology. Consequently, five-axis calibration techniques and dispensing quality detection methods have also attracted considerable attention.

Static error is a major factor affecting machine accuracy and accounts for a large proportion of the total machine error [3]. Geometric error is an important component of the static error [4,5]. The geometric errors of five-axis machine tools are categorized into position-dependent geometric errors (PDGEs) and position-independent geometric errors (PIGEs) [6]. PDGEs are caused by machining and manufacturing defects of the motion axes. PIGEs are produced by assembly errors [7]. The traditional detection and compensation methods primarily rely on measurement methods, such as laser interferometers, ballbars, and R-tests [8,9,10]. The methods enable global offline calibration with micrometer-level accuracy. However, the calibration process has limitations, such as complicated operation, time-consuming, and difficulty with automated self-calibration [11].

In recent years, machine vision technology has offered new approaches to automatic error identification by leveraging its non-contact, integrated, and automated advantages [12,13]. Researchers have achieved high-precision detection of geometric errors through methods such as visual center positioning compensation algorithms combined with self-illuminating collaborative targets, the integration of the product-of-exponential model with adaptive algorithms, and the establishment of spatial reference points [14,15,16]. Geometric error modeling serves as the foundation for identifying and compensating spatial errors in five-axis machine tools [17]. Screw theory is a core mathematical tool for describing rigid body motion and has been widely applied in the kinematic modeling and error analysis of five-axis machine tools [18]. Among various modeling methods, the one based on the local product-of-exponential strikes a good balance between kinematic solution efficiency and error analysis accuracy [15,19], compared with the traditional Denavit–Hartenberg modeling method [20,21] and the classical product-of-exponential model [22,23]. In addition, recent work has begun to explore integrating vision-based calibration and detection functions within the same platform, aiming at unified geometric accuracy evaluation and defect detection [24,25]. From the perspective of in-line quality assurance, dispensing defects such as misalignment, discontinuity, and volume variation are generally detected by automated optical detection. Many existing systems employ rule-based image processing and contour analysis methods, with parameters tuned to specific products and illumination conditions, and can achieve micrometer-level positioning accuracy on PCB-type substrates [26]. Meanwhile, other studies adopt deep-learning-based segmentation and detection networks, as well as multimodal sensing techniques, to identify subtle voids or surface defects in packaging regions, and have shown good performance under complex backgrounds and low defect contrast [27,28,29].

This paper proposes a vision-based measurement (VBM) system for the calibration and detection of a five-axis precision dispensing machine. The kinematic error model of the prototype is established based on the local product-of-exponential model, incorporating geometric error terms into the transformations associated with axis motions. Subsequently, a geometric error calibration algorithm is developed. The target error terms are identified using the axis configuration and motion characteristics of the prototype. A dispensing detection algorithm is designed and applied for adhesive dot position and geometric feature extraction. To improve the efficiency of calibration and detection, a target recognition framework based on the revised random Hough transform (RRHT) algorithm is integrated into the calibration and detection algorithms.

## 2. Design of a VBM-Based Five-Axis Dispensing Machine

### 2.1. Design of a VBM System

**Figure 1:**
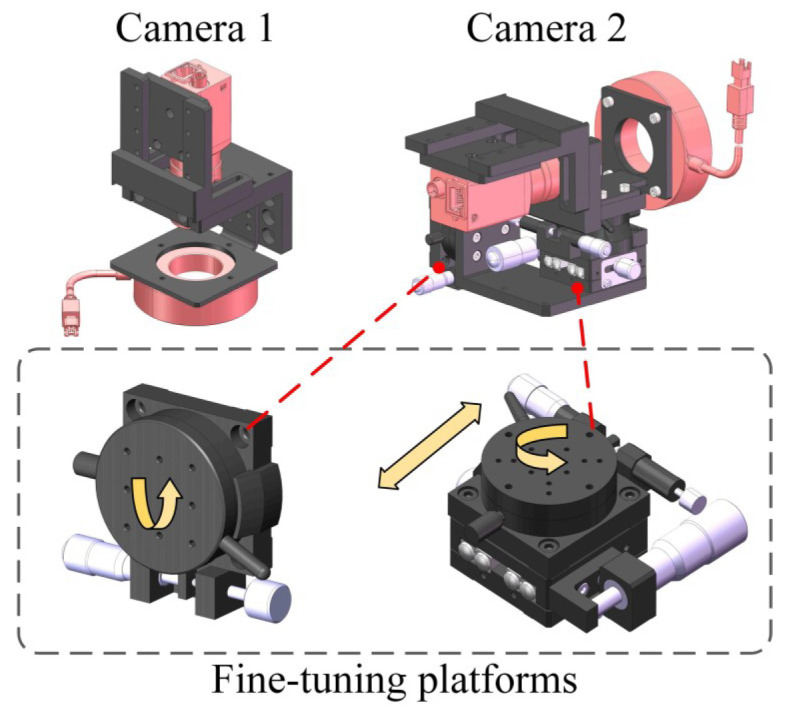
Design of a VBM system using two cameras.

Two cameras are employed to develop a vision-based five-axis system in this paper. Camera 1 is located at the Z-axis to measure the displacements of X-axis, Y-axis, and C-axis, as well as part of the A-axis. Camera 2 is located at the X-axis to measure the A-axis. The hardware and specific structure are shown in Figure 1.

Mounting brackets are designed with an adjustment mechanism to calibrate the spatial position of the system. Camera 2 is mounted on a fine-adjustment mechanism. The position of camera 2 can be adjusted to rotate and translate to optimize the optical axis alignment of the imaging system for A-axis measurement tasks.

### 2.2. Design of a Five-Axis Dispensing Machine Using the VBM System

**Figure 2:**
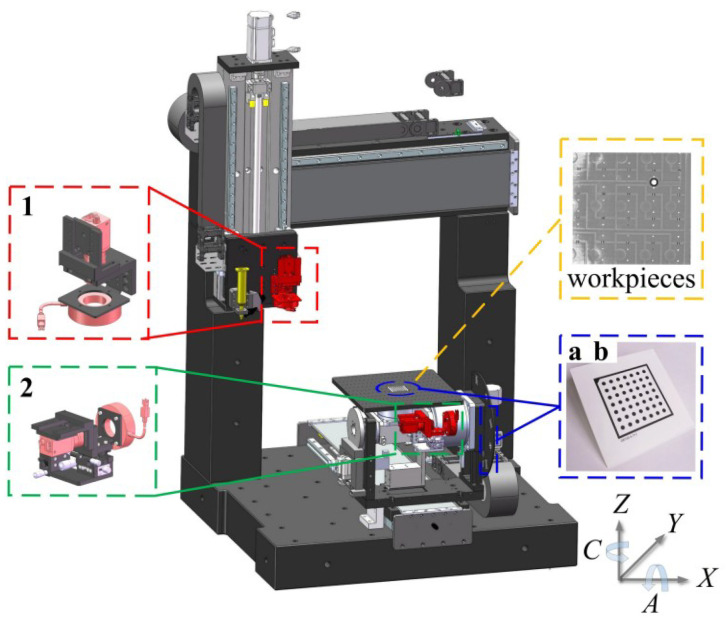
Integration of the VBM system inside a five-axis dispensing machine.

The VBM system is integrated into the five-axis dispensing machine, as shown in Figure 2.

Specifically, camera 1 is mounted on the Z-axis motion module and moves together with the X-axis and Z-axis motion modules. Camera 2 is installed on the A-axis component to measure the A-axis’s related errors and rotates with the component. During the image acquisition process, the calibration features on the calibration board *b* remain within the field of view of camera 2. In calibration, plates *a* and *b* are mounted on the workpiece stage and the A/C-axis bracket, respectively.

The travel parameters of the prototype five-axis dispensing machine used in this study are as follows: the translational stroke of either the X-axis or Y-axis is 600 mm, the Z-axis is 400 mm; the rotational range of the A-axis is ±92°; the rotational range of the C-axis is ±180°.

## 3. Kinematic Identification of the Five-Axis Dispensing Machine Using the VBM System

### 3.1. Kinematic Error Analysis of the Five-Axis Dispensing Machine

**Figure 3:**
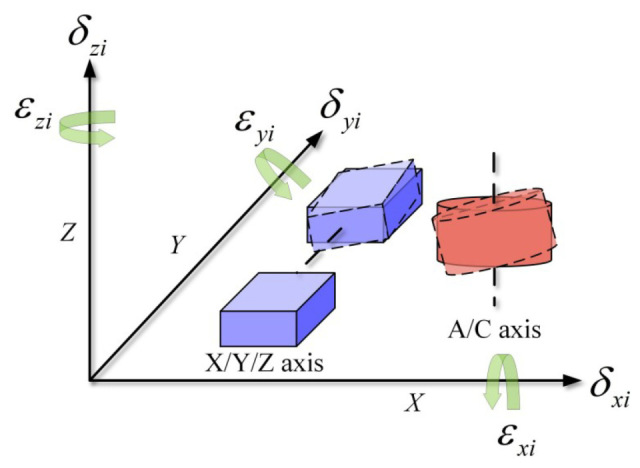
PDGEs of five axes inside the five-axis dispensing machine.

**Figure 4:**
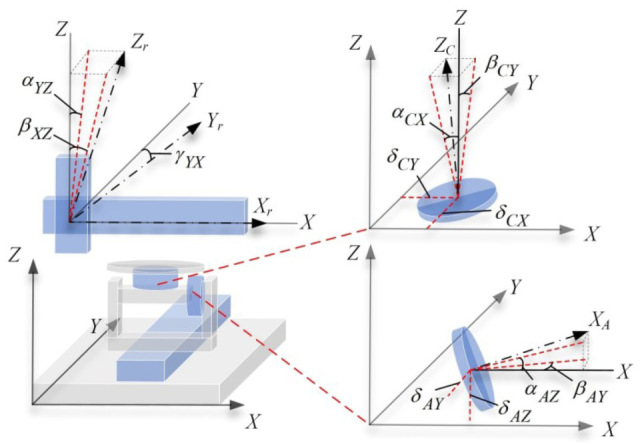
PIGEs of five axes inside the five-axis dispensing machine.

An absolute representation [30] is adopted to define the PDGEs and PIGEs of each axis. As shown in Figure 3 and Figure 4, PDGEs reflect motion-dependent dynamic errors linked to position and angle, while PIGEs represent static geometric defects.

Specifically, the PDGEs are summarized in Table 1, where $\varepsilon_{ii}$ and $\delta_{ii}$ represent the positional error and rotational error of each axis of the five-axis dispensing machine, respectively. PIGEs are summarized in Table 2. Here, $\alpha_{YZ}$, $\beta_{XZ}$, and $\gamma_{YX}$ denote the perpendicularity errors between the X, Y, and Z linear axes; $\alpha_{AZ}$ and $\beta_{AY}$ denote the perpendicularity errors of the A-axis rotation axis relative to the Z-axis and Y-axis, respectively; $\alpha_{CX}$ and $\beta_{CY}$ denote the perpendicularity errors of the C-axis rotation axis relative to the X-axis and Y-axis, respectively; $\delta_{AY}$ and $\delta_{AZ}$ represent the radial errors of the A-axis in the Y-direction and Z-direction, respectively, while $\delta_{CX}$ and $\delta_{CY}$ represent the radial errors of the C-axis in the X-direction and Y-direction, respectively.

### 3.2. Kinematic Error Modeling of the Five-Axis Dispensing Machine

**Figure 5:**
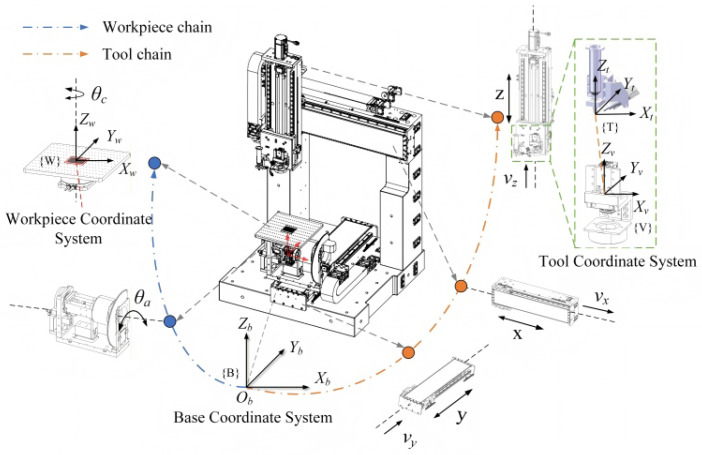
Kinematics chain of the VBM-based five-axis machine.

The local product-of-exponential model is employed for actual forward kinematic modeling of the prototype. The five-axis platform’s actual motion is the product of its ideal motion and the small error motion screw [31]. As shown in Figure 5, when the A/C-axis rotary table of the prototype is at the maximum travel position of the Y-axis motion module, the intersection of the A-axis and C-axis is defined as the base coordinate system $\left\{B\right\}$. The tool coordinate system $\left\{T\right\}$, the camera coordinate system $\left\{V\right\}$, and the workpiece coordinate system $\left\{W\right\}$ are then established to formulate the kinematic chain of the prototype.

The screw coordinate is expressed as follows.

(1) $$\left.{\hat{\xi }}_{i}=\left[\begin{array}{cc} {\hat{\omega }}_{i} & v_{i}\\ 0 & 1 \end{array}\right.\right]\in \mathrm{se}\left(3\right),v_{i}=-\omega \times q_{i}$$

(2) $$\left.{\hat{\omega }}_{i}=\left[\begin{array}{ccc} 0 & -\omega_{i,3} & \omega_{i,2}\\ \omega_{i,3} & 0 & -\omega_{i,1}\\ -\omega_{i,2} & \omega_{i,1} & 0 \end{array}\right.\right]\in \mathrm{so}\left(3\right)$$

where $\omega_{i}$ denotes the unit vector indicating the direction of the rotating joint; $q_{i}$ represents a point lying on the rotation axis.

When $\omega_{i}=0$ in the rotation vector, the angular velocity of motion is zero, and the rigid body only undergoes translational motion. The exponential form of the rotation axis and linear axis is expressed as follows.

(3) $$e^{{\hat{\xi }}_{i}\theta_{i}}=\left\{\begin{array}{c} \begin{array}{c} \left[\begin{array}{cc} e^{{\hat{\omega }}_{i}\theta_{i}}\; & \left(I_{3\times 3}-e^{{\hat{\omega }}_{i}\theta_{i}}\right)\left(\omega_{i}\times \nu_{i}\right)+\omega_{i}\omega_{i}^{T}v_{i}\theta_{i}\\ 0_{1\times 3} & 1 \end{array}\right]\\ \left[\begin{array}{cc} I_{3\times 3} & \theta_{i}v_{i}\\ 0_{1\times 3} & 1 \end{array}\right] \end{array} \end{array}\right.$$

where $e^{{\hat{\omega }}_{i}\theta_{i}}=I_{3\times 3}+{\hat{\omega }}_{i}\sin\theta_{i}+{\hat{\omega }}_{i}^{2}\left(1-\cos\theta_{i}\right)$.

The kinematic chain of five-axis equipment can be divided into two categories: the tool chain and the workpiece chain. By adjusting the arrangement order of kinematic joints, different kinematic chain transformation matrices can be established.

(4) $$\begin{array}{cc} & g_{b}^{t}\left(\theta_{1t},\theta_{2t},\theta_{3t}\ldots \theta_{mt}\right)\\ = & e^{{\hat{\xi }}_{1t}\theta_{1t}}g_{0}^{1}\left(0\right)e^{{\hat{\xi }}_{2t}\theta_{2t}}g_{1}^{2}\left(0\right)\ldots g_{m-1}^{m}\left(0\right)e^{{\hat{\xi }}_{(m+1)t}\theta_{(m+1)t}}g_{m}^{m+1}\left(0\right)\\ = & e^{{\hat{\xi }}_{1t}\theta_{1t}}e^{{\hat{t}}_{0,1}}e^{{\hat{\xi }}_{2t}\theta_{2t}}e^{{\hat{t}}_{1,2}}\ldots e^{{\hat{t}}_{m-1,m}}e^{{\hat{\xi }}_{(m+1)t}\theta_{(m+1)t}}e^{{\hat{t}}_{m,m+1}} \end{array}$$

(5) $$\begin{array}{cc} \; & g_{b}^{w}\left(\theta_{1w},\theta_{2w},\theta_{3w}\ldots \theta_{nw}\right)\\ = & e^{{\hat{\xi }}_{1w}\theta_{1w}}g_{0}^{1}\left(0\right)e^{{\hat{\xi }}_{2w}\theta_{2w}}g_{1}^{2}\left(0\right)\ldots g_{n-1}^{n}\left(0\right)e^{{\hat{\xi }}_{(n+1)w}\theta_{(n+1)w}}g_{n}^{n+1}\left(0\right)\\ = & e^{{\hat{\xi }}_{1w}\theta_{1w}}e^{{\hat{w}}_{0,1}}e^{{\hat{\xi }}_{2w}\theta_{2w}}e^{{\hat{w}}_{1,2}}\ldots e^{{\hat{w}}_{n-1,n}}e^{{\hat{\xi }}_{(n+1)w}\theta_{(n+1)w}}e^{{\hat{w}}_{n,n+1}} \end{array}$$

where $g_{i}^{\;i+1}\left(0\right)\in SE\left(3\right)$(i=1,2,…,n/m) is the initial pose between the coordinate systems; ${\hat{t}}_{i,i+1}\in se\left(3\right)$ and ${\hat{w}}_{i,i+1}\in se\left(3\right)$ represent the Lie algebra form of the transformation matrix $g_{i}^{\;i+1}\left(0\right)$ in the tool chain and the workpiece chain, respectively; ${\hat{\xi }}_{ii}\in se\left(3\right)$ denotes the screw coordinate of the *i* motion axis in the coordinate system, and $\theta_{ii}$ denotes the joint displacement of axis *i*.

The kinematic model of the prototype system used in this work can be expressed.

(6) $$\begin{array}{cc} \; & g_{t}^{w}\left(\theta_{a},\theta_{c},\theta_{y},\theta_{x},\theta_{z}\right)={\left[g_{b}^{w}\left(\theta_{a},\theta_{c}\right)\right]}^{-1}g_{b}^{t}\left(\theta_{y},\theta_{x},\theta_{z}\right)\\ = & e^{-{\hat{w}}_{c,w}}e^{-{\hat{\xi }}_{c}\theta_{c}}e^{-{\hat{w}}_{a,c}}e^{-{\hat{\xi }}_{a}\theta_{a}}e^{-{\hat{w}}_{b,a}}e^{{\hat{t}}_{b,y}}e^{{\hat{\xi }}_{y}\theta_{y}}e^{{\hat{t}}_{y,x}}e^{{\hat{\xi }}_{x}\theta_{x}}e^{{\hat{t}}_{x,z}}e^{{\hat{\xi }}_{z}\theta_{z}}e^{{\hat{t}}_{z,v}}e^{{\hat{t}}_{v,t}} \end{array}$$

The error propagation model of the PDGEs can be expressed as follows.

(7) $$\begin{array}{c} e^{{\hat{\xi }}_{i}^{D}\theta_{i}}=e^{{\hat{\xi }}_{\delta_{xi}}\delta_{xi}}e^{{\hat{\xi }}_{\delta_{yi}}\delta_{yi}}e^{{\hat{\xi }}_{\delta_{zi}}\delta_{zi}}e^{{\hat{\xi }}_{\varepsilon_{xi}}\varepsilon_{xi}}e^{{\hat{\xi }}_{\varepsilon_{yi}}\varepsilon_{yi}}e^{{\hat{\xi }}_{\varepsilon_{zi}}\varepsilon_{zi}} \end{array}$$

The error propagation model is derived by multiplying the corresponding motion matrices of the PIGEs. Accordingly, the transfer matrix of the PIGEs associated with the A-axis and C-axis rotary shafts can be obtained.

(8) $$e^{{\hat{\xi }}_{a}^{I}\theta_{a}}=e^{{\hat{\xi }}_{\delta_{AZ}}\delta_{AZ}}e^{{\hat{\xi }}_{\delta_{AY}}\delta_{AY}}e^{{\hat{\xi }}_{\alpha_{AZ}}\alpha_{AZ}}e^{{\hat{\xi }}_{\beta_{AY}}\beta_{AY}}$$

(9) $$e^{{\hat{\xi }}_{c}^{I}\theta_{c}}=e^{{\hat{\xi }}_{\delta_{CX}}\delta_{CX}}e^{{\hat{\xi }}_{\delta_{CY}}\delta_{CY}}e^{{\hat{\xi }}_{\beta_{CY}}\beta_{CY}}e^{{\hat{\xi }}_{\alpha_{CX}}\alpha_{CX}}$$

Since the X-axis is the reference axis, the Y-axis and Z-axis represent the translational axis perpendicularity error transformation matrix.

(10) $$e^{{\hat{\xi }}_{y}^{I}\theta_{y}}=e^{{\hat{\xi }}_{\gamma_{YX}}\left(-\gamma_{YX}\right)}$$

(11) $$e^{{\hat{\xi }}_{z}^{I}\theta_{z}}=e^{{\hat{\xi }}_{\alpha_{YZ}}\alpha_{YZ}}e^{{\hat{\xi }}_{\beta_{XZ}}\left(-\beta_{XZ}\right)}$$

The actual forward kinematic model of the five-axis dispensing machine can be obtained.

(12) $$\begin{array}{cc} {\tilde{g}}_{t}^{w}= & e^{-{\hat{w}}_{c,w}}\left(e^{{\hat{\xi }}_{c}^{D}\theta_{c}}e^{{\hat{\xi }}_{c}^{I}\theta_{c}}e^{-{\hat{\xi }}_{c}\theta_{c}}\right)e^{-{\hat{w}}_{a,c}}\left(e^{{\hat{\xi }}_{a}^{D}\theta_{a}}e^{{\hat{\xi }}_{a}^{I}\theta_{a}}e^{-{\hat{\xi }}_{a}\theta_{a}}\right)e^{-{\hat{w}}_{b,a}}\\ \; & e^{{\hat{t}}_{b,y}}\left(e^{{\hat{\xi }}_{y}^{D}\theta_{y}}e^{{\hat{\xi }}_{y}^{I}\theta_{y}}e^{{\hat{\xi }}_{y}\theta_{y}}\right)e^{{\hat{t}}_{y,x}}\left(e^{{\hat{\xi }}_{x}^{D}\theta_{x}}e^{-{\hat{\xi }}_{x}\theta_{x}}\right)e^{{\hat{t}}_{x,z}}\left(e^{{\hat{\xi }}_{z}^{D}\theta_{z}}e^{{\hat{\xi }}_{z}^{I}\theta_{z}}e^{-{\hat{\xi }}_{z}\theta_{z}}\right)e^{{\hat{t}}_{z,v}}e^{{\hat{t}}_{v,t}} \end{array}$$

### 3.3. Geometric Error Identification

This section identifies the PDGEs and PIGEs contained in the error model based on a stepwise error identification method. Both the A-axis and C-axis are located in the workpiece chain, and the error propagation model of the workpiece chain can be derived from Equation (12).

(13) $$\begin{array}{c} {\left[e^{-{\hat{w}}_{c,w}}\right]}^{-1}{\tilde{g}}_{b}^{w}\left(\theta_{a},\theta_{c}\right){\left[e^{-{\hat{w}}_{b,a}}\right]}^{-1}{\left[g_{b}^{w}\left(0\right)\right]}^{-1}=e^{{\hat{\xi }}_{a}^{I}\theta_{a}}e^{-{\hat{w}}_{a,c}}e^{{\hat{\xi }}_{c}^{I}\theta_{c}} \end{array}$$

where ${\tilde{g}}_{b}^{w}\left(\theta_{a},\theta_{c}\right)$ denotes the motion transformation matrix of the workpiece relative to the base coordinate system, and $g_{b}^{w}\left(0\right)$ represents the ideal initial pose matrix.

(14) $$\begin{array}{cc} \delta_{CX} & ={\tilde{R}}_{b}^{w}\left(1,4\right),\delta_{AZ}={\tilde{R}}_{b}^{w}\left(3,4\right),\delta_{AY}+\delta_{CY}={\tilde{R}}_{b}^{w}\left(2,4\right)\\ \beta_{AY} & ={\tilde{R}}_{b}^{w}\left(2,1\right),\beta_{CY}={\tilde{R}}_{b}^{w}\left(3,2\right),\alpha_{CX}+\alpha_{AZ}={\tilde{R}}_{b}^{w}\left(1,3\right) \end{array}$$

where ${\tilde{R}}_{b}^{w}\left(n,m\right)$ denotes the element in the *n* row and *m* column of the matrix product on the left side of Equation (13).

When $\theta_{a}=0$, only the C-axis moves, and the corresponding pose matrix can be obtained.

(15) $${\left[e^{{\hat{\xi }}_{a}^{I}\theta_{a}}e^{{\hat{\xi }}_{c}^{I}\theta_{c}}\right]}^{-1}{\left[e^{-{\hat{w}}_{c,w}}\right]}^{-1}g_{b}^{w}\left(0,\theta_{c}\right){\left[e^{{\hat{\xi }}_{c}\theta_{c}}g_{b}^{w}\left(0\right)\right]}^{-1}=e^{{\hat{\xi }}_{c}^{D}\theta_{c}}$$

(16) $$\left\{\begin{array}{c} \varepsilon_{yc}=\arcsin\;\left({\tilde{R}}_{bc}^{w}\left(1,3\right)\right)\\ \varepsilon_{xc}=-\arcsin\;\left(\frac{{\tilde{R}}_{bc}^{w}\left(2,3\right)}{\cos(\varepsilon_{yc})}\right)\\ \varepsilon_{zc}=-\arcsin\;\left(\frac{{\tilde{R}}_{bc}^{w}\left(1,2\right)}{\cos(\varepsilon_{yc})}\right) \end{array}\right.$$

(17) $${\left[\begin{array}{ccc} \delta_{xc} & \delta_{yc} & \delta_{zc} \end{array}\right]}^{T}={\left[\begin{array}{ccc} {\tilde{R}}_{bc}^{w}\left(1,4\right) & {\tilde{R}}_{bc}^{w}\left(2,4\right) & {\tilde{R}}_{bc}^{w}\left(3,4\right) \end{array}\right]}^{T}$$

where ${\tilde{R}}_{bc}^{w}\left(n,m\right)$ denotes the element in the *n* row and *m* column of the matrix product on the left side of Equation (15).

When $\theta_{c}=0$, only the A-axis moves, and the pose matrix can be expressed.

(18) $$\begin{array}{c} {\left[e^{{\hat{\xi }}_{a}^{I}\theta_{a}}e^{{\hat{\xi }}_{c}^{I}\theta_{c}}\right]}^{-1}{\left[e^{-{\hat{w}}_{c,w}}\right]}^{-1}g_{b}^{w}\left(\theta_{a},0\right){\left[e^{{\hat{\xi }}_{a}\theta_{a}}g_{b}^{w}\left(0\right)\right]}^{-1}=e^{{\hat{\xi }}_{a}^{D}\theta_{a}} \end{array}$$

(19) $$\left\{\begin{array}{c} \varepsilon_{ya}=\arcsin\;\left({\tilde{R}}_{ba}^{w}\left(1,3\right)\right)\\ \varepsilon_{xa}=-\arcsin\;\left(\frac{{\tilde{R}}_{ba}^{w}\left(2,3\right)}{\cos(\varepsilon_{ya})}\right)\\ \varepsilon_{za}=-\arcsin\;\left(\frac{{\tilde{R}}_{ba}^{w}\left(1,2\right)}{\cos(\varepsilon_{ya})}\right) \end{array}\right.$$

(20) $${\left[\begin{array}{ccc} \delta_{xa} & \delta_{ya} & \delta_{za} \end{array}\right]}^{T}={\left[\begin{array}{ccc} {\tilde{R}}_{ba}^{w}\left(1,4\right) & {\tilde{R}}_{ba}^{w}\left(2,4\right) & {\tilde{R}}_{ba}^{w}\left(3,4\right) \end{array}\right]}^{T}$$

where ${\tilde{R}}_{ba}^{w}\left(n,m\right)$ denotes the element in the *n* row and *m* column of the matrix product on the left side of Equation (18).

The error propagation model of the tool chain can be derived from Equation (12).

(21) $$\begin{array}{cc} {\tilde{g}}_{b}^{t}= & e^{{\hat{t}}_{b,y}}\left(e^{{\hat{\xi }}_{y}^{D}\theta_{y}}e^{{\hat{\xi }}_{y}^{I}\theta_{y}}e^{{\hat{\xi }}_{y}\theta_{y}}\right)e^{{\hat{t}}_{y,x}}\left(e^{{\hat{\xi }}_{x}^{D}\theta_{x}}e^{-{\hat{\xi }}_{x}\theta_{x}}\right)e^{{\hat{t}}_{x,z}}\left(e^{{\hat{\xi }}_{z}^{D}\theta_{z}}e^{-{\hat{\xi }}_{z}\theta_{z}}\right)e^{{\hat{t}}_{z,v}}e^{{\hat{t}}_{v,t}} \end{array}$$

Then, the perpendicularity error of the translation axis is calculated using the components of the actual motion vector v.

(22) $$\left\{\begin{array}{cc} \gamma_{YX} & =acos\left(v_{x}\cdot v_{y}\right)-\frac{\pi }{2}\\ \alpha_{YZ} & =acos\left(v_{y}\cdot v_{z}\right)-\frac{\pi }{2}\\ \beta_{XZ} & =acos\left(v_{x}\cdot v_{z}\right)-\frac{\pi }{2} \end{array}\right.$$

## 4. Calibration and Detection Using the VBM System

### 4.1. Design of a RRHT-Based Target Detection Framework

To improve the accuracy and efficiency of the VBM system in detecting circular dot calibration boards and dispensed workpieces, this study proposes an RRHT-based target detection framework, which consists of three modules: image preprocessing, edge detection, and target recognition. In preprocessing, median filtering and gamma correction are introduced to handle noise and illumination variations. For edge detection, the Canny algorithm is used to extract contours. The target recognition phase consists of four steps: circle parameter calculation, candidate circle screening, real circle determination, and least squares fitting.

**Figure 6:**
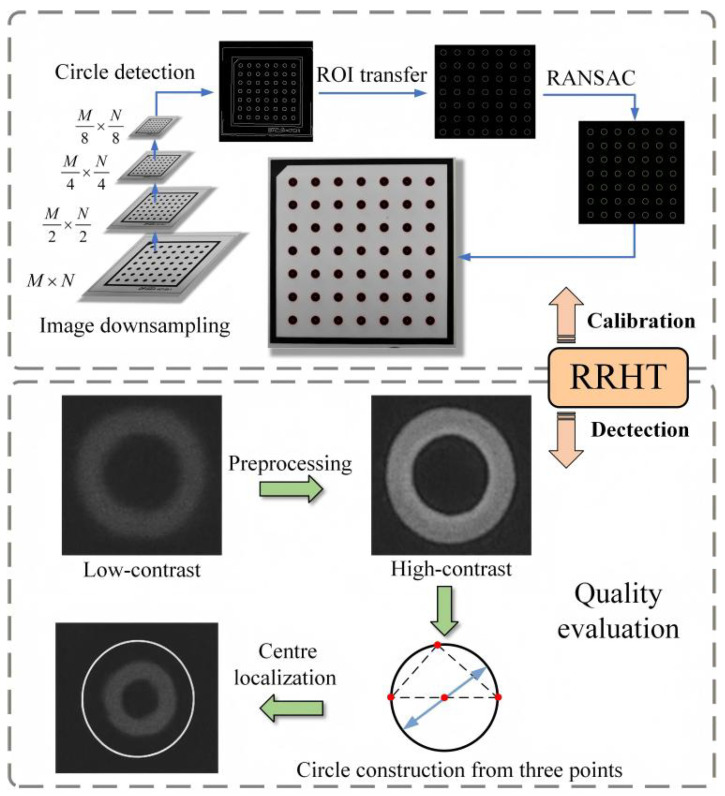
Principle of the RRHT algorithm.

In the circle parameter calculation stage, the algorithm adopts a pixel-gradient-based sampling strategy to improve efficiency, thereby overcoming the excessive computational load of traditional random sampling. To control parameter sensitivity and ensure robustness against noise, a gradient threshold is applied to filter invalid edge points before sampling. In the candidate circle screening and true circle determination stages, the algorithm calculates the ratio of the number of pixels on the circle to its theoretical circumference. This ratio serves as a dynamic constraint for the accumulation threshold. The method effectively minimizes false detections under varying lighting conditions [32]. Finally, the least-squares method is applied to fit the detected true circles, further enhancing recognition accuracy. The specific steps are illustrated in Figure 6.

Adaptation and optimization schemes were applied to two targets: the dot calibration grid and the dispensed workpiece. For the grid with regularly arranged circular dots, post-processing downsampling reduced computation, while Freeman chain codes encoded edges to remove non-circular noise. Circular ROIs were fitted using RANSAC to minimize information loss. For the dense annular adhesive dot with low contrast, contrast-limited adaptive histogram equalization enhanced edges. After edge detection, noise and adhesion were filtered by contour length and convexity. Circular ROIs minimized misalignment, and the effective pixel ratio along the ring edge identified valid adhesive dots. Finally, sub-pixel center localization and dot quality evaluation were performed using least squares.

### 4.2. Design of a Vision-Based Five-Axis Calibration Algorithm

Based on the kinematic model and error identification strategy established in Section 3, a vision-based error calibration algorithm is designed. The algorithm leverages the Eigen library for matrix arithmetic and the GiNaC library for symbolic computation.

**Figure 7:**
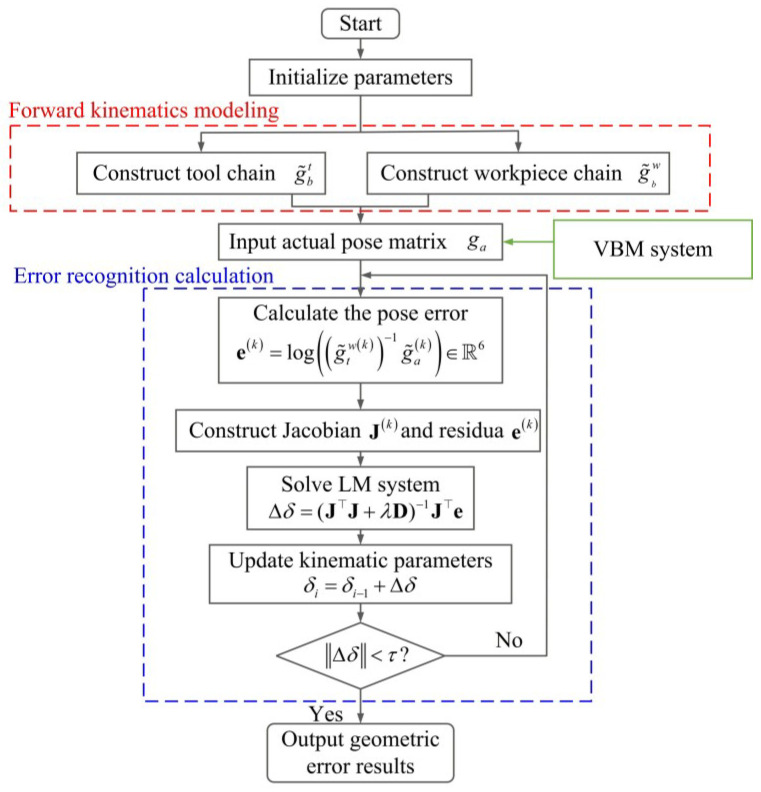
Strategy of the error identification algorithm.

As shown in Figure 7, the algorithm starts by initializing structural parameters to define the spatial relationship between the camera and the target. To improve computational efficiency, closed-form transformation functions are employed, minimizing the need for trigonometric and matrix operations compared to traditional screw exponential mappings. Fixed and repeated transformations are cached to avoid redundancy. Based on Equations (4) and (5), the pose matrices ${\tilde{g}}_{b}^{t}$ and ${\tilde{g}}_{b}^{w}$ for the tool and workpiece chains are constructed, and the theoretical pose ${\tilde{g}}_{t}^{w}$ is calculated via Equation (12). The VBM system captures an image of the calibration board, and then the Perspective-n-Point (PnP) algorithm is used to estimate the actual pose $g_{a}$. The algorithm measures the pose error on the special Euclidean group SE(3) using the logarithmic map. For the *k*-th sample, the residual is expressed as

(23) $$e^{\left(k\right)}=\log\;\left(\;{\left({\tilde{g}}_{t}^{w(k)}\right)}^{-1}{\tilde{g}}_{a}^{\left(k\right)}\right)\in R^{6}.$$

The residuals corresponding to *N* different poses of the calibration board (N=300 in this study) are stacked vertically. For the unknown error parameter vector δ, the Jacobian matrix J=∂e/∂δ is computed for each sample and concatenated vertically. In implementation, J is obtained using a numerically stable finite-difference approximation. The Levenberg–Marquardt (LM) algorithm is employed to optimize the error-compensation parameters iteratively:

(24) $$\Delta \delta ={\left(J^{⊤}J+\lambda \;D\right)}^{-1}J^{⊤}e.$$

where λ is the damping factor and D is the diagonal matrix of $J^{\;⊤}J$, and Δδ is the error parameter compensation amount for the current iteration step. In this study, the convergence threshold is set as $\tau =10^{-5}$.

To address the potential ill-conditioning of the Jacobian matrix, singular value decomposition and regularization techniques are incorporated into the solution process to enhance robustness, and the optimal estimates of the error terms are eventually obtained.

### 4.3. Design of a Detection Strategy for the Dispensed Workpiece

**Figure 8:**
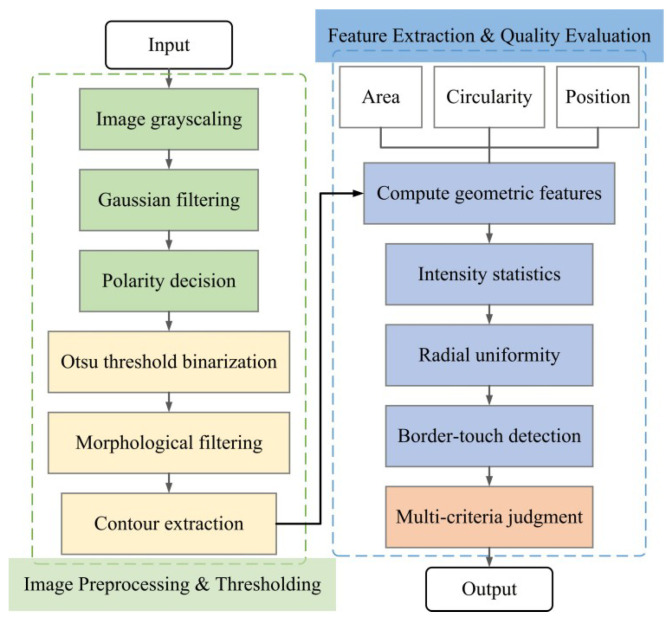
Design of a detection algorithm.

To ensure the consistency of adhesive dot morphology and the reliability of defect detection during the dispensing process, a dispensing detection algorithm is proposed. The algorithm takes a grayscale image as input and first applies Gaussian smoothing for noise suppression. Considering that adhesive dots may appear as either bright or dark foregrounds in the image, an automatic polarity discrimination mechanism is designed. It simultaneously generates positive and negative thresholding results, performs morphological opening and closing to suppress noise and repair edges, and automatically determines the optimal polarity according to a scoring criterion that prioritizes the number of valid contours and secondarily the total target area. The algorithm is shown in Figure 8.

After obtaining the optimal binary result, candidate regions are extracted subject to area constraints, and multi-dimensional features are computed to characterize both geometric and photometric properties of the adhesive dots. The geometric features include area *A*, perimeter *P*, diameter *d*, and circularity *C*, which describe the integrity and regularity of the adhesive dot shape.

To further quantify the contour uniformity, the radius relative to the centroid $\left(\bar{x},\bar{y}\right)$ is defined as

(25) $$r_{i}=\sqrt{{\left(x_{i}-\bar{x}\right)}^{2}+{\left(y_{i}-\bar{y}\right)}^{2}}$$

where $\left(x_{i},y_{i}\right)$ denotes the coordinates of contour points.

The mean and standard deviation of the radius are calculated.

(26) $$\mu_{r}=\frac{1}{N}\sum_{i}r_{i},\;\sigma_{r}=\sqrt{\frac{1}{N}\sum_{i}{\left(r_{i}-\mu_{r}\right)}^{2}}$$

where *N* is the number of contour points.

The normalized standard deviation of radius is then defined.

(27) $$NSD_{r}=\frac{\sigma_{r}}{\mu_{r}}$$

This scale-invariant metric evaluates the circumferential uniformity of the contour, where a smaller $NSD_{r}$ indicates a more circular and uniform shape.

For intensity uniformity evaluation, the mean $\mu_{I}$ and standard deviation $\sigma_{I}$ of pixel intensities within the segmented mask are computed, and the coefficient of variation of intensity is used to quantify internal grayscale uniformity:

(28) $$CoV_{I}=\frac{\sigma_{I}}{\mu_{I}}$$

A smaller $CoV_{I}$ indicates a more homogeneous grayscale distribution within the adhesive dot region.

The final decision is made according to a threshold set $\left\{A,C,\tau_{r},\tau_{I}\right\}$, while excluding regions that touch the image boundary. The classification criteria are expressed.

(29) $$\left\{\begin{array}{cc} A_{\min} & \leq A\leq A_{\max},\\ C & \geq C_{\min},\\ NSD_{r} & \leq \tau_{r},\\ CoV_{I} & \leq \tau_{I} \end{array}\right.$$

Targets satisfying all constraints are classified as qualified; otherwise, they are considered defective.

## 5. Test of the VBM System Inside the Five-Axis Dispensing Machine

### 5.1. Prototype Fabrication and Test Setup

**Figure 9:**
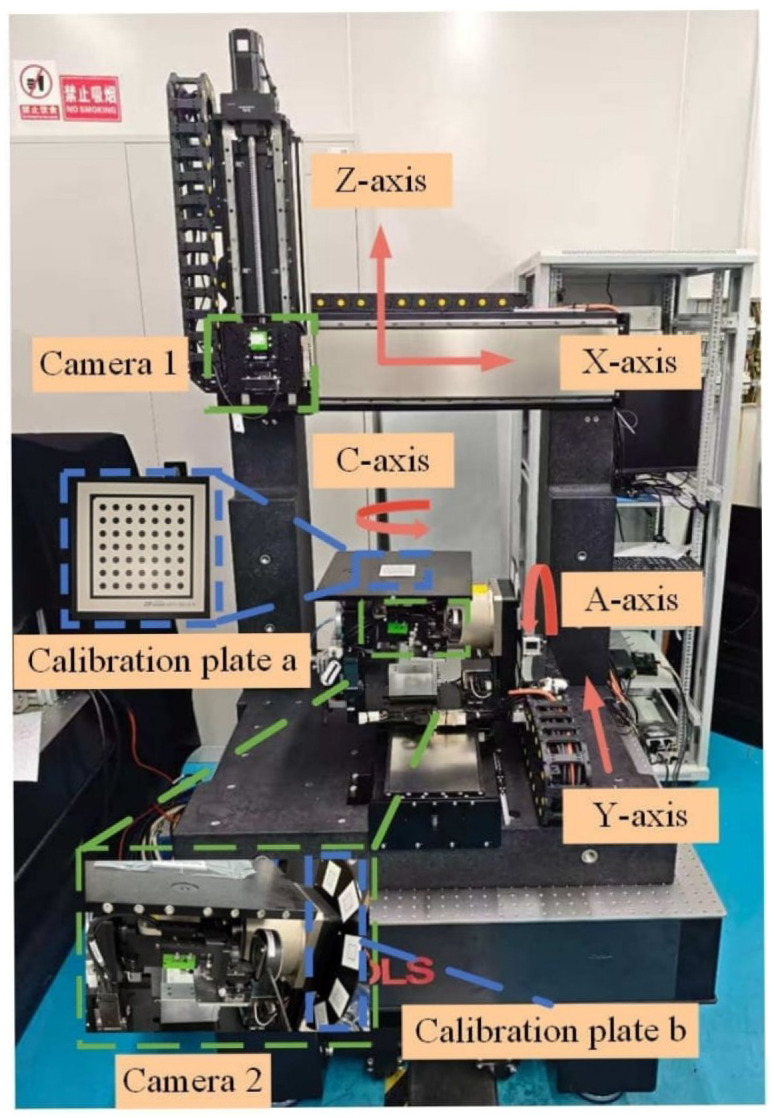
Test setup of the VBM system inside the five-axis dispensing machine.

For this experimental VBM-based prototype, the motion controller is SPiiPlusEC (ACS, Israel), which supports multi-axis coordinated motion; the vision system consists of two cameras (OPT-CC1-M060-GR1-10, 3072 × 2048 pixels, OPT, China), two lenses (OPT-COB1628, OPT, China), two ring light sources (RI7000-W, OPT, China), and two types of standard dot calibration plates (Array 7×7, circle diameters of 1.0 mm and 2.5 mm, respectively, with an accuracy of 0.1 μm, DFvision, China). The experimental setup is shown in Figure 9.

### 5.2. Test of the Kinematic Identification and Calibration Using the VBM System

Prior to the measurement experiments, ten independent self-calibration trials were conducted for camera 1 and camera 2, respectively. Each trial used a distinct set of 15 calibration images captured at different poses. The reprojection errors of all trials and their mean values are summarized in Table 3. Meanwhile, the magnitudes of the five distortion parameters are below 0.4 across all trials. The self-calibration accuracy satisfies the requirements for subsequent measurements.

The performance of the RRHT-based target detection framework in estimating the pose of calibration plates, together with its runtime and recognition accuracy compared to the Blob-based framework and the Hough-based framework, is shown in Table 4. The RRHT-based framework exhibits advantages in recognition efficiency. Although its reprojection error is slightly higher, the RRHT algorithm offers a better trade-off between speed and accuracy for automated calibration tasks with large datasets.

To evaluate the performance of the kinematic modeling method (proposed in Section 3.2) in the error calibration algorithm, all other modules were kept unchanged in the experiment, and only this module was replaced. Under this configuration, the mean single-run time of each method, the total time required to construct 300 sets of error matrices, and the error norms were calculated. The results are presented in Table 5.

The results indicate that the kinematic model based on the local product-of-exponential exhibits certain advantages in terms of runtime and modeling accuracy compared with the Denavit–Hartenberg modeling method and the global product-of-exponential model; in contrast, there are no significant differences in these two indicators between the proposed method and the local product-of-exponential model (presented in Reference [15]), where geometric errors are embedded into the initial pose matrices of adjacent coordinate systems. A noticeable performance gap is observed only for the global product-of-exponential formulation, possibly due to its simplified assumption of fixed global screw axes in the presence of joint coupling.

**Figure 10:**
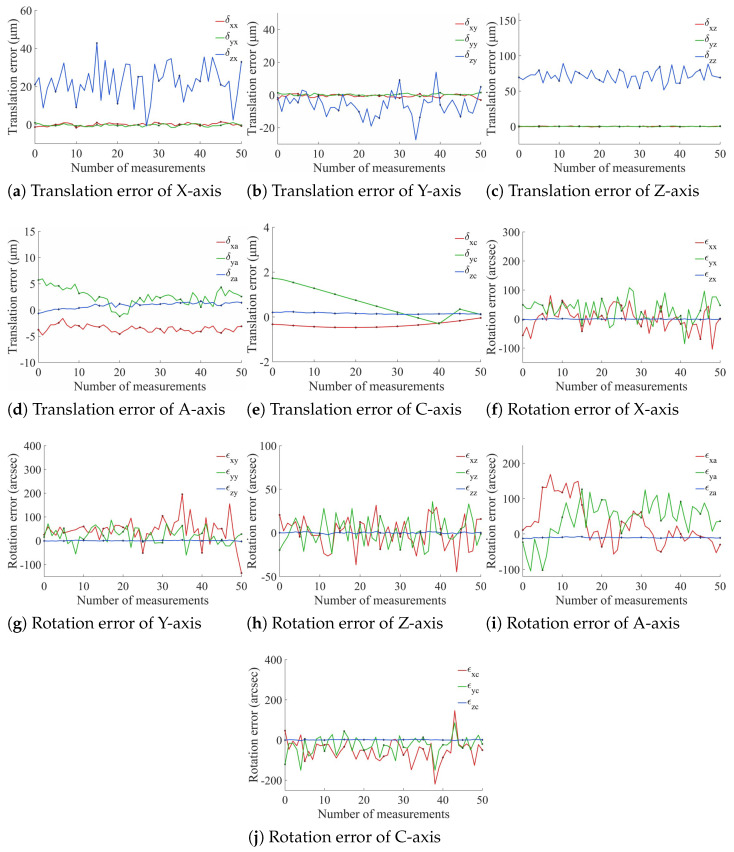
Measurement results of PDGEs.

During the experiment, the X-axis and Y-axis moved in 1 mm increments, the Z-axis moved in 0.1 mm increments, and the rotary axes rotated in 1° increments. Fifty sets of experimental data were collected during the motion process, with each set recorded five times. The VBM system was used to measure and analyze the calibration plate image set, and the mean values were taken to ultimately obtain the PDGE measurement results for each axis, as shown in Figure 10.

**Figure 11:**
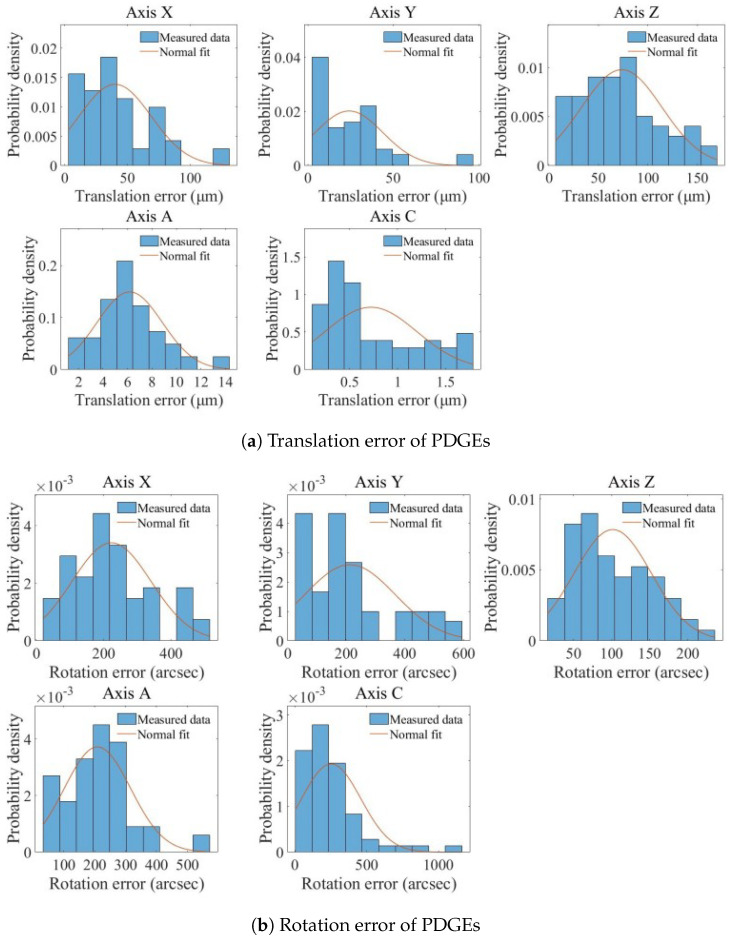
Identification results of PDGEs.

Based on the measurement results shown in Figure 10, the statistical distributions of the translational and rotational PDGEs for the five axes are derived and shown in Figure 11. For the translational errors, the three linear axes exhibit mean values of 40.2 μm (X), 24.8 μm (Y), and 73.5 μm (Z). The two rotary axes show more compact distributions, with mean values of 6.18 μm (A) and 0.720 μm (C). The rotational errors exhibit more pronounced differences in magnitude: the mean values for the X-axis, Y-axis, and A-axis are 224″, 213″, and 209″, respectively; the Z-axis has a mean of 101″; and the C-axis spans a wider range with a mean of 253″. Overall, the Z-axis translational error is relatively larger, which may be related to changes in camera focal distance during upward movement along the Z-direction. The errors of all axes are distributed around their respective mean values with varying degrees of dispersion, and the empirical histograms show good consistency with the fitted normal distributions. Machining errors exist on each axis. Since the motion axes are driven by ball screws or direct-drive motors, their precision of motion is inherently limited. Furthermore, servo-induced tracking delays or overshoots can contaminate the measured error profiles.

Based on the error identification strategy, sampling experiments were conducted encompassing both single-axis and five-axis modes. The study completed the eight PIGEs for the A-axis and C-axis, with the error identification results shown in Table 6. The five-axis dispensing machine used in this experiment integrates axes from different manufacturers. The linear axes are mounted on a marble gantry frame, providing a stable installation reference. The assembly accuracy of the rotary axes primarily depends on the manufacturing and assembly precision of the machined components produced by turning and milling machines, thus exerting a more significant influence on PIGEs.

The identification results focus on the eight PIGEs of the A-axis and C-axis. The proposed method can be further extended by incorporating additional geometric error terms into the kinematic model together with appropriate measurement strategies. For the linear axes, the squareness errors among the X-axis, Y-axis, and Z-axis can be calculated based on the identified screw coordinates and are obtained from the angles between the corresponding motion direction vectors.

**Figure 12:**
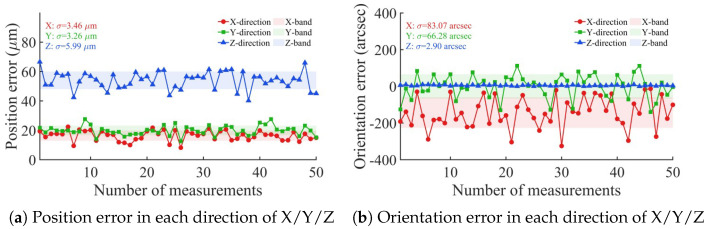
Single direction error in X/Y/Z direction before calibration.

PIGEs and PDGEs are reflected in the position and orientation accuracy of the end position of the dispensing tip, as measured by the VBM system and shown in Figure 12. The mean position and orientation errors over *m* validation poses are defined.

(30) $$\Delta t=\frac{1}{m}\sum_{i=1}^{m}∥\;t_{a,i}-t_{c,i}\;∥,$$

(31) $$\Delta R=\frac{1}{m}\sum_{i=1}^{m}∥\;\log\;{\left(R_{a,i}^{-1}R_{c,i}\right)}^{\vee }∥,$$

where $t_{a,i}$ and $t_{c,i}$ are the measured and calculated position vectors of the dispensing tip, $R_{a,i}$ and $R_{c,i}$ are the corresponding rotation matrices.

From the results, it can be seen that the position error lies within the single direction of X/Y/Z before calibration is in the range of 0.0 μm∼70.0 μm. The standard deviations are 3.46 μm, 3.26 μm, and 5.99 μm for the X, Y, and Z directions, respectively. The orientation error in the X-direction before calibration varies the most in the range of −360″∼0.0″, followed by the Y-direction in the range of −180″∼180″, and the Z-direction is the smallest in the range of −360″∼ 360″. The corresponding standard deviations of orientation error are 83.07″, 66.28″, and 2.90″ for the X, Y, and Z directions, respectively.

**Figure 13:**
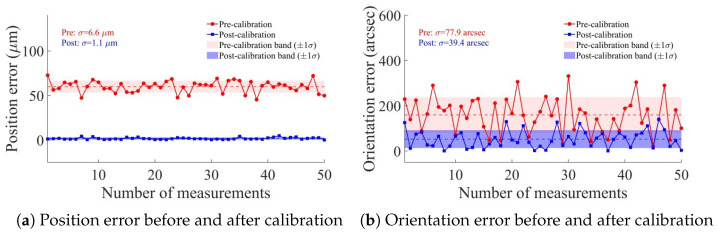
Position and orientation error before and after calibration.

The compensation values for each axis were calculated using an inverse kinematics algorithm, which was then applied to compensate for the motion of each axis. The error results after running the algorithm are shown in Figure 13.

From the algorithm-level analysis, the mean position error is reduced from 59.9 μm to 1.80 μm, the mean orientation error is reduced from 160″ to 52.9″, the maximum position error is reduced from 72.7 μm to 4.90 μm, and the maximum orientation error is reduced from 331″ to 140″. Furthermore, the standard deviation of the position error was suppressed from 6.6 μm to 1.1 μm, and the orientation error standard deviation decreased from 77.9″ to 39.4″. The theoretical improvement rate of position error is 96.1%, and that of orientation error is 67.4%. Residual errors in the system primarily stem from VBM inaccuracies and kinematic modeling limitations. Measurement precision is constrained by camera calibration uncertainties, including lens distortion and focal length drift during Z-axis motion, as well as image noise. Furthermore, the model’s small-error assumption neglects higher-order terms, while certain unmodeled geometric errors may persist as systematic residuals after compensation.

### 5.3. Test of the Detection Using the VBM System

**Figure 14:**
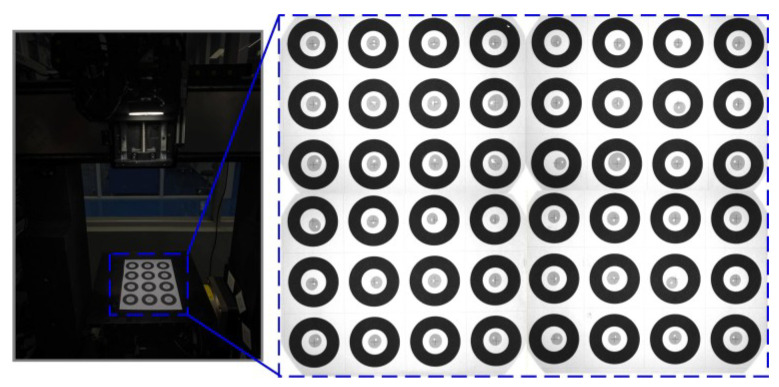
Adhesive test sample.

A preliminary verification was conducted on the dispensing detection algorithm described in Section 4.3, testing its measurement performance for three characteristics of UV adhesive dots: position accuracy, roundness, and equivalent diameter. Some adhesive dot samples are shown in Figure 14.

**Figure 15:**
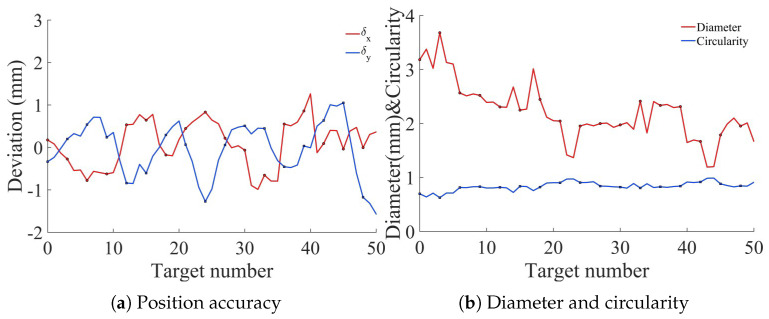
Adhesive dot detection results.

During the test, UV adhesive dots were applied within a black circular ring target. Taking the ring center as the theoretical center $\left(x_{0},y_{0}\right)$ and the adhesive dot center as the actual center (x,y), their X-direction deviation $\delta_{x}$ and Y-direction deviation $\delta_{y}$ served as the reference standard for position accuracy. The adhesive dot detection results are shown in Figure 15.

The adhesive dot detection experiment was conducted on 50 samples acquired under a fixed illumination and imaging configuration. The proposed detection algorithm showed stable detection performance across all samples. The average values of $\delta_{x}$ and $\delta_{y}$ were 0.04 mm and −0.06 mm, respectively. Furthermore, the algorithm is able to extract geometric features, including diameter and circularity, which were consistent with the actual sample morphology. These results demonstrate the feasibility of the method for low-contrast UV adhesive applications under controlled conditions, serving as a preliminary exploration of the calibration effectiveness in practical applications.

## 6. Conclusions and Future Work

This study focuses on the geometric error identification and vision-based detection of a five-axis precision dispensing machine, proposing a set of error modeling and calibration methods based on VBM, which have been verified under experimental conditions. The main conclusions are summarized as follows:

1. A five-axis precision dispensing machine integrated with a VBM system was designed, and its kinematics were modeled using a local product-of-exponential formulation. This model features a relatively concise formulation and reliably computes the kinematic chain pose within the experimental range. The mean calculation time for a single set of data is approximately 0.088 ms, showing slight advantages in efficiency and accuracy compared with the Denavit–Hartenberg modeling method.
2. A vision-based five-axis geometric error calibration algorithm was designed, which can be used to measure and identify the PDGEs of each axis and the eight items of PIGEs of the A-axis and C-axis of the prototype. Comprehensive measurements obtained using the VBM system show that the end-effector exhibits a mean spatial position error of approximately 59.9 μm and a mean orientation error of about 160 arcsec, representing the measured geometric error level of the prototype. For the VBM system, an RRHT-based target detection framework was designed to improve its measurement efficiency.
3. A dispensing detection algorithm was preliminarily verified using sample adhesive dots. The proposed pipeline achieves reliable position and geometric feature extraction (diameter, circularity) of the adhesive dots. The current experiments, however, cover a limited number of samples and dot types, so the present results mainly demonstrate the basic applicability of the method rather than a comprehensive performance evaluation.

Future work will focus on further improving the measurement accuracy and stability of the VBM system, including repeatability experiments based on extended datasets, as well as more uncertainty analysis, enhancing the precision of geometric error measurement and identification, and completing the comprehensive recognition of PIGEs and PDGEs for all five axes. In addition, comparative studies with established industrial inspection solutions will be considered to further evaluate the proposed framework in practical application scenarios. Meanwhile, continuous optimization of the dispensing detection algorithm will be pursued through extended experimental validation and algorithm refinement, by incorporating deep learning and intelligent image analysis techniques to improve adaptability and robustness under complex operating conditions.

## Figures and Tables

**Table 1:** PDGEs inside the five-axis dispensing machine.

X-Axis	Y-Axis	Z-Axis	A-Axis	C-Axis
$\varepsilon_{xx}$	$\varepsilon_{xy}$	$\varepsilon_{xz}$	$\varepsilon_{xa}$	$\varepsilon_{xc}$
$\varepsilon_{yx}$	$\varepsilon_{yy}$	$\varepsilon_{yz}$	$\varepsilon_{ya}$	$\varepsilon_{yc}$
$\varepsilon_{zx}$	$\varepsilon_{zy}$	$\varepsilon_{zz}$	$\varepsilon_{za}$	$\varepsilon_{zc}$
$\delta_{xx}$	$\delta_{xy}$	$\delta_{xz}$	$\delta_{xa}$	$\delta_{xc}$
$\delta_{yx}$	$\delta_{yy}$	$\delta_{yz}$	$\delta_{ya}$	$\delta_{yc}$
$\delta_{zx}$	$\delta_{zy}$	$\delta_{zz}$	$\delta_{za}$	$\delta_{zc}$

**Table 2:** PIGEs inside the five-axis dispensing machine.

PIGEs for Translational Axes	PIGEs for Rotary Axes	
$\alpha_{YZ}$	$\delta_{AY}$	$\delta_{CX}$
$\beta_{XZ}$	$\delta_{AZ}$	$\delta_{CY}$
$\gamma_{YX}$	$\alpha_{AZ}$	$\alpha_{CX}$
	$\beta_{AY}$	$\beta_{CY}$

**Table 3:** Camera self-calibration results of the VBM system.

Image Group		1	2	3	4	5	6	7	8	9	10	Avg.
Reprojection error (pixel)	Camera 1	0.165	0.244	0.141	0.205	0.212	0.162	0.157	0.150	0.115	0.173	0.172
	Camera 2	0.182	0.191	0.160	0.169	0.174	0.127	0.222	0.155	0.130	0.169	0.168

**Table 4:** Performance of the target recognition framework with the PnP algorithm integration.

Item	RRHT	Blob	Hough
Reprojection error (pixel)	0.897	0.620	0.664
Detection time (s)	10.5	28.8	20.9
Pose estimation time (s)	4.36	4.37	4.44

**Table 5:** Comparison of Algorithm Modules Based on Different Kinematic Modeling Methods.

Modeling Method	Mean Time/Group (ms)	Total Time (ms)	Error Norm
Denavit–Hartenberg [33]	0.116	33.2	0.138
Global product-of-exponential [23]	0.230	69.9	1.023
Local product-of-exponential [15]	0.089	26.8	<0.001
Proposed method	0.088	25.3	<0.001

**Table 6:** Identification results of PIGEs.

PIGEs	$\alpha_{\mathrm{AZ}}$	$\beta_{\mathrm{AY}}$	$\alpha_{\mathrm{CX}}$	$\beta_{\mathrm{CY}}$
Values (arcsec)	−1234.8	−263.9	−986.4	2142.0
PIGEs	$\delta_{AY}$	$\delta_{AZ}$	$\delta_{CX}$	$\delta_{CY}$
Values (μm)	−5.13	456	241	705

## Data Availability

The raw data supporting the conclusions of this article will be made available by the authors on request.
